# Comparison of psychiatric disability on the health of nation outcome scales (HoNOS) in resettled traumatized refugee outpatients and Danish inpatients

**DOI:** 10.1186/s12888-014-0330-8

**Published:** 2014-12-18

**Authors:** Sabina Palic, Michelle Lind Kappel, Monica Stougaard Nielsen, Jessica Carlsson, Per Bech

**Affiliations:** National Centre for Psychotraumatology, University of Southern Denmark, Odense, Denmark; Clinic for PTSD and Transcultural Psychiatry (CPTP), Aarhus University Hospital, Aarhus, Denmark; Competence Center for Transcultural Psychiatry Denmark, Psychiatric Center Ballerup, The Mental Health Services of the Capital Region of Denmark, Copenhagen, Denmark; Psychiatric Research Unit, Psychiatric Center North Zealand, Hillerød, Denmark

**Keywords:** Psychiatric disability, HoNOS, Refugee, Trauma, Treatment

## Abstract

**Background:**

Currently, the mental health issues of traumatized refugees are mainly documented in terms of posttraumatic stress disorder, depression, and anxiety. Importantly, there are no reports of the level of psychiatric disability in treatment seeking traumatized refugees resettled in the West. Insufficient acknowledgment of the collective load of bio-psycho-social problems in this patient group hinders effective psychiatric and social service utilization outside the specialized clinics for traumatized refugees.

**Methods:**

The level of psychiatric disability in traumatized refugees from Danish specialized clinics (N = 448) is documented using routine monitoring data from pre- and post-treatment on the Health of Nation Outcome Scales (HoNOS). Furthermore, the HoNOS ratings are compared with routine monitoring data from Danish inpatients with different diagnoses (N = 10.911).

**Results:**

The routinely collected data indicated that despite their outpatient status, traumatized refugees had higher levels of psychiatric disability at pre-treatment compared to most inpatients. Moreover, the traumatized refugees had a HoNOS profile characterized by an overall high problem level in various psychiatric and social domains. The rate of pre- to post-treatment improvement on the HoNOS was smaller for the traumatized refugees than it was for the psychiatric inpatients.

**Conclusions:**

The level, and the versatile profile, of psychiatric disability on the HoNOS point to complex bio-psycho-social problems in resettled treatment seeking traumatized refugees. Thus, a broader assessment of symptoms and better cooperation between psychiatric, health care, and social systems is necessary in order to meet the treatment needs of this group.

## Background

Treatment-seeking refugees resettled in the West experience many stressors before their arrival at a host country [1]. They are often exposed to multiple traumas before and during migration, and many also experience post-settlement difficulties such as language barriers, culture shock, and loss of social status and social networks [2]. These stressors place refugees at high risk of developing different psychiatric disorders. In current research, the mental health issues experienced by traumatized refugees in the West are often interpreted in the light of a few psychiatric disorders, the most common being posttraumatic stress disorder (PTSD), depression, and anxiety [3]. In clinical settings, a range of other problems are typically encountered, however, they are often difficult to document as there are only a few validated measures available for the assessment of this group [4]. There are currently no validated or commonly applied measures for the combined load of biopsychosocial problems in traumatized refugees [4]. Consequently, the present understanding of the complex conditions experienced by treatment seeking traumatized refugees resettled in the West is limited. Moreover, as refugees often seek treatment for trauma-related problems many years after having resettled in the West, most are rightly considered to be former refugees (with permanent residence or citizenship) by the time they encounter Western psychiatric systems. Thus, traumatized *former* refugees constitute a subgroup of psychiatric patients in Western countries whose needs are generally poorly understood and poorly documented.

Psychiatric disability is often defined as the sum of impairments in the biological, psychological, and social areas of functioning [5]. This type of global bio-psycho-social evaluation is inherent in most areas of Western psychiatric care. This is because the development of psychiatric disability is often related to different risk and protective factors than those related to the development of specific psychiatric symptoms. Also, recovery from psychiatric disability is usually known to lag behind that of symptoms of specific mental disorders [6]. Hence, a systematic assessment of psychiatric disability in addition to the symptoms of specific mental disorders is important in guiding individual, as well as political decisions about treatment needs and prognosis in different groups of psychiatric patients.

To the best of our knowledge, there are currently no studies that describe the level of psychiatric disability in representative groups of traumatized refugees from Western clinics. One small study analyzed 50 Health of Nation Outcome Scales (HoNOS) case files from refugees and asylum seekers in London community psychiatry, and found that these service users had greatly elevated levels of psychiatric disability compared to users without refugee experiences [7]. Documentation of psychiatric disability in traumatized refugees in larger groups with better representativity is needed to improve cooperation between specialized refugee clinics and other parts of the social and psychiatric systems, especially when it comes to the facilitation of a much needed mutual understanding of the overall severity of psychiatric symptoms and social problems in this treatment seeking population. In particular, a wider acknowledgement of the level of psychiatric disability in traumatized treatment seeking refugees in the West is important within systems that make decisions about the possibility of recovery and societal participation after treatment in specialized refugee clinics.

In the present study, the level of psychiatric disability in traumatized refugees from Danish specialized clinics is documented using routine Health of Nation Outcome Scales (HoNOS) monitoring data from pre- and post-treatment. Furthermore, in order to facilitate the understanding of the level of psychiatric disability beyond the walls of the specialized clinics for traumatized refugees, the HoNOS ratings are compared with routine monitoring data from Danish inpatients with different diagnoses. Specific problems experienced by traumatized refugees are highlighted on the HoNOS profile, and rates of improvement for each group are compared in order to aid understanding about disability and prognosis. Finally, perspectives on the practical use of the HoNOS in Western clinics for traumatized refugees are offered.

## Method

HoNOS ratings for traumatized refugee patients from three departments (Aarhus, Horsens, Randers) of the Clinic for PTSD and Transcultural Psychiatry (CPTP), Aarhus University Hospital, were routinely collected at intake and discharge over a 3-year period. CPTP is a specialized center for the treatment of trauma and torture within the psychiatric services of the Danish mental health system. The comparison group consisted of psychiatric inpatients at the Psychiatric Center North Zealand (PCNZ), which is a general psychiatric hospital within the Danish mental health system. HoNOS ratings for psychiatric inpatients at PCNZ were collected routinely at intake and discharge over a 10-year period [8,9].

### Refugee treatment setting and procedures

All traumatized refugee patients who started and finished treatment at one of the three CPTP departments during the period of May, 2009 to April, 2012 were eligible participants. Individuals are referred to CPTP by social workers and general practitioners when exposure to refugee experiences and war trauma are suspected to be the main cause of their psychiatric problems. Moreover, individuals must have a diagnosis of one or more of the following ICD-10 [10] disorders in order to qualify for treatment: *depressive disorders* (F32-34), *anxiety disorders* (F40-49), and, *Enduring Personality Change after Catastrophic Experience* (F62.0). Individuals with possible traumatization who also fulfill the criteria for a primary *psychotic disorder* and/or severe *substance abuse* are not eligible for treatment at CPTP and are referred to other treatments. The CPTP offers outpatient, multidisciplinary treatment, including weekly psychotherapy and physiotherapy, as well as counseling in relation to psychoactive medication and social issues. The average length of treatment for the traumatized refugees in the present study was approximately 4 months (M = 130.5 days, SD = 56.61). Demographic information and HoNOS ratings (at pre- and post-treatment) were obtained by the psychologists. The traumatized refugees were rated on the HoNOS by the same psychologist who provided psychotherapy. The first HoNOS administration had to be completed by the fourth treatment session at the latest (including one session of general pre-treatment assessment). Only individuals with established refugee status are treated at the CPTP. Thus, the present group does not comprise any asylum seekers.

### Psychiatric inpatient setting and procedures

Individuals who were inpatients at PCNZ for more than 24 hours during the years 2000 to 2009 were included in the comparison group [8,9]. The psychiatric inpatients were diagnosed according to the ICD-10 [10]. All major diagnostic groups were represented in the comparison group (see Table 1). The inpatients received standard psychiatric treatment according to their condition. Length of admission also varied according to condition. Further details about the treatment can be found in Bech et al. [11]. The HoNOS was administered by psychiatric nurses no longer than 24 hours after intake and again at time of discharge. Patients who were admitted to the hospital several times during the course of one year feature only once in the present data. The study was approved by Aarhus University Hospital and PCNZ according to ethical rules for data collected as a part of routine monitoring. Informed consent is not requested from the patients when routinely collected data are analyzed retrospectively for research purposes.

**Table 1:** **Demographic information**

	**Refugee outpatients**	**Schizophrenia**	**Affective disorders**	**All anxiety disorders**	**Personality disorders**	**Addiction**	**Dementia**
		**(F20-29)**	**(F30-39)**	**(F40-49)**	**(F60-69)**	**(F10-19)**	**(F00-09)**
	**N = 448**	**N = 3257**	**N = 3111**	**N = 1818**	**N = 1061**	**N = 994**	**N = 670**
**Gender (% female)**	45.5	46.3	62.4	62.8	73.7	38.8	44.2
**Age in years**	40.5	42.6	55.6	41.5	38.0	46.5	65.7
**(SD; range)**	(8.57; 20-61)	(14.3; 18-94)	(16.5; 18-95)	(15.1; 18-94)	(13.1; 18-91)	(13.4; 18-94)	(19.4; 18-94)
**Country of origin** ^**a**^ **- N (%)**							
The Balkans			116 (25.90)				
The Middle East			291 (64.95)				
Other			39 (8.7)				
**Years of formal educationin country of origin** ^**b**^							
0			38 (9)				
1-5			54 (12.8)				
6-10			157 (37.2)				
> 10			173 (41)				
**Trauma history (mutually overlapping groups)**							
Imprisonment			160 (35.71)				
Torture			140 (31.25)				
War			417 (93.10)				
**Need of interpreter** ^**c**^			284 (63.4)				
**Mean length of resettlement** ^**d**^ **(SD, range)**			12.5 years				
			(6.14, 0-29)				

^a^Balkans (Kosovo, Bosnia & Herzegovina, Serbia, Georgia, Montenegro, Macedonia, Croatia), Middle East (Iraq, Iran, Lebanon, Jordan, Kuwait, Syria, Yemen, Afghanistan), Other (Somalia, Sri Lanka, Indonesia, Congo, Vietnam, Ethiopia, Colombia), ^b^n = 422, ^c^n = 445, ^d^n = 439.

### The measure

The *Health of the Nation Outcome Scales* (HoNOS) was developed for routine monitoring of psychiatric patients in the National Health Service of the United Kingdom [12]. It is an observer-rated scale that covers psychological symptoms as well as behavioral, organic and social problems. The HoNOS has been employed and validated across a variety of psychiatric populations in England and other Western countries - including Denmark [11]. It has good concurrent, content, and predictive validity, as well as adequate inter-rater reliability and sensitivity to change [13]. The HoNOS is considered to have psychometric properties corresponding to, or even better than, other psychiatric, observer-rated routine measures [13], of which the Global Assessment of Functioning (GAF) [14] is probably the best known. Each item on the HoNOS is scored on a scale of 0–4 by a clinical observer. Higher scores indicate greater levels of impairment. Scores on the HoNOS can be summed up to produce a total score, reflecting the overall level of psychiatric disability. Scores of ≥ 2 within specific areas indicate levels of impairment that require clinical attention [11]. Ratings of comorbidity (item 8) for the inpatients in the present study were a priori limited to anxiety. Ratings of comorbidity for the traumatized refugees were not limited to any particular disorder (thus followed standard HoNOS rating procedure).

### Data analyses

Statistical analyses were performed using SPSS for Windows 20.0 (SPSS Inc.). The highest percentage of missing data (at the variable level) was 16% for the traumatized refugee patients. Little’s MCAR test indicated that data were missing completely at random. Missing HoNOS data were imputed using the Expectation Maximation Algorithm [15] in SPSS. Data for inpatients were not imputed. Data are described as frequencies and percentages. Differences in pre-treatment HoNOS scores between the traumatized refugees - and psychiatric inpatient groups were assessed using independent sample t-tests. Due to the large sample sizes the critical p-value was set at p = .01. Furthermore, Bonferonni adjustments for 78 repeated tests (corresponding to 13 x 6 comparisons) were made. Effectively, this meant that only tests with p < .0001 were considered significant. As even small differences tend to become significant in large sample sizes, the clinical significance of the differences in pre-treatment HoNOS scores between the traumatized refugee patients- and each of the psychiatric inpatient groups is also reported as Cohen’s d/standardized effect sizes (ES), corrected for unequal group size [16]. Generally speaking, ES = 0.2 indicates a small effect size (i.e. 85% overlap in scores between the two groups), ES = 0.5 indicates a medium effect size (i.e. 67% overlap in scores between the two groups), and ES = 0.8 indicates a large effect size (i.e.53% overlap in scores between the two groups).

## Results

Approximately 93% of all eligible traumatized refugee outpatients and 90% of the psychiatric inpatients participated in the study. Demographic characteristics of the traumatized refugee outpatients and groups of psychiatric inpatients are presented in Table 1. Information about years of formal education was unavailable for inpatients.

### Profiles of psychiatric disability at pre-treatment

Table 2 presents descriptive statistics and highlights significant comparisons between the traumatized refugee outpatients and all other groups on the HoNOS. Table 3 presents standardized effect sizes (ES) that correspond to the observed differences. According to Table 2 the average total HoNOS severity rating for the traumatized refugees was high in comparison to the psychiatric inpatients. The HoNOS profile of the traumatized refugees further revealed that they were rated by the clinicians as needing clinical attention on three HoNOS domains; *depressed mood*, *comorbidity*, and *problems with relationships*. The profiles of psychiatric inpatients typically had fewer areas requiring clinical attention, which were primarily reflective of core problems in their respective diagnoses.

**Table 2:** **Means, standard deviations, and significant comparisons for HoNOS ratings**

**HoNOS item**	**Refugee outpatients**	**Schizophrenia**	**Affective disorders**	**All anxiety disorders**	**Personality disorders**	**Addiction**	**Dementia**
		**(F20-29)**	**(F30-39)**	**(F40-49)**	**(F60-69)**	**(F10-19)**	**(F00-09)**
	**n = 448 (SD)**	**n = 3175 (SD)**	**n = 3081 (SD)**	**n = 1781 (SD)**	**n = 1030 (SD)**	**n = 950 (SD)**	**n = 950 (SD)**
Behavioural problems							
1 Overactive, aggression	1.14 (0.82)	0.67* (1.04)	0.39* (0.81)	0.36* (0.78)	0.49* (0.89)	0.63* (1.04)	1.09 (1.27)
2 Non-accidental self-injury	0.46 (0.71)	0.34 (0.85)	0.50 (1.06)	0.79* (1.29)	0.74* (1.19)	0.59 (1.13)	0.24* (0.75)
3 Problem-drinking or drug taking	0.12 (0.47)	0.64* (1.14)	0.47* (0.97)	0.55* (1.03)	0.74* (1.18)	2.73* (1.11)	0.34* (0.91)
Organic problems							
4 Cognitive problems	1.60 (0.76)	0.76* (1.01)	0.52* (0.87)	0.29* (0.67)	0.36* (0.71)	0.82* (1.11)	2.14* (1.36)
5 Physical illness	1.70 (0.98)	0.52* (0.99)	0.79* (1.12)	0.62* (1.07)	0.56* (0.97)	0.87* (1.17)	1.54 (1.40)
Psychological problems							
6 Hallucinations/delusions	0.60 (1.0)	2.10* (1.30)	0.48 (0.97)	0.32* (0.77)	0.57 (1.0)	0.73 (1.17)	1.38* (1.32)
7 Depressed mood	**2.20** (0.84)	0.88* (0.88)	1.87* (1.11)	1.48* (0.93)	1.42* (0.91)	1.13* (0.96)	0.93* (0.96)
8 Comorbidity/Anxiety	**2.90** (0.92)	2.09* (1.10)	1.96* (1.10)	2.04* (1.10)	2.11* (1.03)	1.87* (1.17)	2.06* (1.26)
Social problems							
9 Problems with relationships	**2.10** (0.93)	1.58* (1.27)	1.04* (1.15)	1.02* (1.17)	1.43* (1.18)	1.27* (1.23)	1.66* (1.43)
10 Problems with activities and…	1.30 (0.83)	1.11* (1.16)	0.95* (1.11)	0.52* (0.92)	0.76* (1.02)	1.06* (1.22)	2.00* (1.48)
11 Living conditions	0.60 (0.92)	0.71 (1.14)	0.41* (0.90)	0.56 (1.15)	0.55 (1.05)	0.78 (1.24)	0.98* (1.37)
12 Problems with occupation	0.95 (1.0)	0.77 (1.12)	0.52* (0.94)	0.47* (0.95)	0.52* (0.92)	0.83 (1.18)	1.19 (1.43)
Total	15.70 (5.60)	12.16* (5.87)	9.90* (5.19)	9.01* (4.96)	10.24* (5.17)	13.32* (6.54)	15.55 (7.32)

Note: HoNOS = Health of Nation Outcome Scales, *p < .0001, clinically important elevations are bolded, because of the number of comparisons, t-values are not reported (can be acquired through the first author).

**Table 3:** **Effect sizes (Cohen’s d) of the pre-treatment differences on the HoNOS between refugee outpatients and every other group**

**HoNOS item**	**Schizophrenia**	**Affective disorders**	**All anxiety disorders**	**Personality disorders**	**Addiction**	**Dementia**
	**(F20-29)**	**(F30-39)**	**(F40-49)**	**(F60-69)**	**(F10-19)**	**(F00-09)**
1 Overactive, aggression	.46	.92	.99	.75	.53	.05
2 Non-accidental self-injury	.14	-.04	-.28	-.28	-.13	.35
3 Problem-drinking /drug taking	-.48	-.38	-.46	-.62	-2.8	-.29
4 Cognitive problems	.86	1.3	1.9	1.7	.78	-.47
5 Physical illness	1.2	.82	1.0	1.2	.74	.12
6 Hallucinations or delusions	-1.2	.12	.34	.03	-.12	-.65
7 Depressed mood	1.5	.31	.80	.88	1.2	1.4
8 Comorbidity/Anxiety	.75	.87	.80	.79	.94	.75
9 Problems with relationships	.42	.94	.96	.61	.73	.35
10 Problems with activities and daily living	.17	.33	.87	.56	.22	-.56
11 Living conditions	-.10	.21	.04	.05	-.16	-.32
12 Problems with occupation	.16	.45	.49	.45	.11	-.19
Total	.60	1.1	1.31	1.0	.38	.02

*Note*: HoNOS = Health of Nation Outcome Scales, effect sizes with minus in front indicate lower ratings of refugee outpatients than the reference group.

#### Behavioral domain

The traumatized refugee patients were rated significantly *higher* than most groups of psychiatric inpatients on *overactive and aggressive behavior-* with differences corresponding to medium to large ES’s. But they acquired significantly *lower* scores on *non-accidental self-injury* and *drinking/drug taking problems* compared to most inpatient groups (ES = 0.48 – 2.8).

#### Organic problems

The traumatized refugees had significantly *lower* clinician-rated scores than inpatients with dementia on *cognitive problems* (ES = .47), but significantly *higher* clinician-rated scores than other groups of psychiatric inpatients (medium to very large ES’s). Regarding *physical illness,* the traumatized refugees had significantly *higher* clinician-rated scores than all groups of psychiatric inpatients apart from those with dementia. Group differences represented large to very large ES’s.

#### Psychological problems

As can be expected, the traumatized refugee outpatients had significantly *lower* clinician-rated scores than the inpatients with schizophrenia and dementia on *hallucinations and delusions* (large to very large ES’s). However, they had significantly *higher* scores than all groups of psychiatric inpatients on *problems with depressed mood* (large to very large ES’s, except those for affective disorders; ES = 0.31), and they displayed significantly *higher* levels of comorbidity/anxiety than all psychiatric inpatients (representing large or close to large ES’s). The most prevalent comorbidities for refugee outpatients were anxiety (31%), hyper-vigilance related to PTSD (25%), and sleep disturbance (16%).

#### Social problems

The traumatized refugees had significantly *higher* clinician rated scores than all groups of psychiatric inpatients on *problems with relationships* (group differences ranging from small to large ES). Significant differences in scores *on problems with activities and daily living* were found between traumatized refugee outpatients and all groups of psychiatric inpatients, with refugees having significantly *higher* clinician-rated scores than all inpatients apart from those with dementia (ES = .17-.87). With regards to ratings *on problems with living conditions*, few statistically significant differences with small ES’s were found between the traumatized refugees and psychiatric inpatients. Traumatized refugee outpatients had significantly higher scores on *occupational problems* than patients with affective-, anxiety-, and personality disorders (medium sized ES’s).

Finally, the total level of psychiatric disability of the traumatized refugee outpatients was found to be significantly *higher* than that for inpatients with schizophrenia, addiction, affective- , anxiety- and personality disorders, and approximately at the same level as that of inpatients with dementia. In terms of clinical importance, the mean difference in total HoNOS scores between traumatized refugees and inpatients with addiction and schizophrenia were small and medium sized, respectively. However, the traumatized refugee patients obtained considerably higher total HoNOS severity ratings compared to inpatients with affective-, anxiety-, and personality disorders (ES = 1.0 to 1.31).

### The rate of pre- to post-treatment improvement on the HoNOS

Table 4 indicates that the overall rate of pre- to post-treatment improvement on the HoNOS for traumatized refugees was 10%. The improvement rates for psychiatric inpatients ranged between 23 to 49%. The traumatized refugee outpatients showed the largest improvements in relation to *behavioral problems* and some *psychological symptoms*. The psychiatric inpatients demonstrated a greater all-round improvement. All groups showed less improvement in relation to *social problems* compared to other areas of impairment.

**Table 4:** **Percentage of improvement from pre-treatment to post-treatment on the health of nation outcome scales (HoNOS)**

**HoNOS item**	**Refugee out-patients**	**Schizophrenia**	**Affective disorders**	**All anxiety disorders**	**Personality disorders**	**Addiction**	**Dementia**
		**(F20-29)**	**(F30-39)**	**(F40-49)**	**(F60-69)**	**(F10-19)**	**(F00-09)**
1 Overactive, aggression	18%	65%	63%	62%	47%	69%	54%
2 Non-accidental self-injury	32%	81%	83%	77%	71%	70%	74%
3 Problem-drinking or drug taking	0%	45%	57%	45%	40%	36%	47%
4 Cognitive problems	11%	31%	32%	35%	29%	34%	9%
5 Physical illness	0%	17%	16%	22%	22%	24%	5%
6 Hallucinations or delusions	2%	47%	69%	59%	57%	72%	49%
7 Depressed mood	18%	53%	61%	53%	52%	54%	39%
8 Comorbidity/Anxiety	10%	47%	54%	46%	42%	49%	35%
9 Problems with relationships	14%	23%	42%	32%	22%	26%	15%
10 Problems with activities and daily living	8%	25%	40%	37%	25%	29%	5%
11 Living conditions	5%	20%	30%	17%	16%	19%	9%
12 Problems with occupation	3%	24%	30%	23%	11%	21%	10%
Total	10%	39%	49%	44%	38%	40%	23%

## Discussion

The present findings indicate *higher* overall levels of psychiatric disability among resettled traumatized refugee outpatients compared to most psychiatric inpatients, who by definition are in the most acute phase of their psychiatric disorders. Thus, the clinician rated level of psychiatric disability in treatment seeking traumatized refugees is shown to correspond more closely to that of the severe psychiatric inpatients with schizophrenia, dementia, and addiction, and considerably less to that of inpatients with affective-, anxiety-, and personality disorders.

However, due to the present study design, which utilized routinely collected data, contextual factors in the treatment settings should be considered as potential contributors to the findings. Namely, in the present study, the traumatized refugee outpatients were compared to psychiatric inpatients. The best comparison to the traumatized refugees would have been outpatients with affective and/or anxiety disorders without refugee experiences, but HoNOS ratings were not available for these groups. In this case, large representative groups of inpatients encompassing a range of the most frequent psychiatric diagnoses (including those with affective and anxiety disorders) were used as a comparison.

There are arguments both for and against this approach. What speaks against this approach is that ratings for inpatients were made by psychiatric nurses and those for the traumatized refugees were made by psychologists. - These two groups of professionals occupy different roles in the treatment, and have different sources of information available for the rating of the HoNOS. - One can in general assume that in an inpatient setting, the ratings are on the whole more influenced by *observations* of behavior, while those in an outpatient setting are to a higher degree *inferred* through conversations *about* behavior. Thus, these two types of ratings are not necessarily the same. However, one can also argue that they do not have to be inherently different either. The psychiatric nurses are those professionals who spend the most time with the inpatients during their hospitalization. So, as in the case of the psychologists, their ratings are probably based on their overall knowledge of the patients, including their conversations with them, and other routine assessments, that they performed at the PCNZ.

What speaks in favor of the comparison between the traumatized refugee outpatients and psychiatric inpatients is the fact that HoNOS is made for purposes of comparing outcomes across different treatments [12]. Prior studies had thus indicated that it is able to differentiate between inpatients and outpatients [17], and that clinical psychologists in general do not tend to make systematic overratings on the HoNOS when compared to psychiatric nurses and doctors [12]. Finally, very similar levels of disability on the HoNOS have been reported for asylum seekers and refugees within community treatment in London [7]. Hence, the fact that similar severity levels on the HoNOS have been reported for traumatized refugees across outpatient treatments in different European countries makes it less probable that systematic overrating took place at CPTP.

In sum, the advantage of this study is that it presents representative data directly from the clinical practice, and documents the *everyday* disability evaluations of large patient groups (and especially those of the traumatized refugees). However, there is no way to directly test the possible influence of the inpatient vs. outpatient treatment in a retrospective, naturalistic design such as the present. Future studies documenting psychiatric disability in treatment seeking traumatized refugees on the HoNOS should therefore aim at including other outpatients as well.

If one accepts the proposition that traumatized refugees in Western outpatient treatment have very high levels of psychiatric disability, a number of characteristics associated with the refugee experience itself can help explain the findings. The average length of resettlement in Denmark and the time at which treatment was sought by traumatized refugees in this study was very long (M = 12 years). This may have contributed to the chronicity of symptoms of psychiatric illnesses among the traumatized refugees, and, consequently higher levels of social impairment. Moreover, the very high levels of disability found among traumatized refugee outpatients are probably also linked to the risk factors associated with the experience of being a refugee. Namely, individuals who, aside from having a psychiatric illness, also display severe problems with societal participation and social networks (i.e. have social impairment) are by definition considered to be disabled [5]. In this respect, refugees are particularly vulnerable, because they are often exposed to additional social challenges associated with migration and various post-settlement difficulties. A 10-year follow-up study of tortured, treatment-seeking refugees found that post-settlement difficulties had a negative effect on psychiatric morbidity, and that this effect increased over time [18]. Thus, given that refugees generally have few protective factors such as social support, employment, and societal inclusion, and that those who develop trauma-related psychiatric disorders tend to seek treatment at a late stage, the finding of very high levels of psychiatric disability in treatment seeking traumatized refugees is not so surprising.

With regards to the specific profile of disability on the HoNOS, the present findings indicate that the HoNOS scores obtained by the traumatized refugees are clearly discernible only from those obtained by the inpatients with schizophrenia, dementia, and addiction. Apart from this, refugee outpatients often received higher or equally high ratings on the core problem areas of other diagnostic groups. Importantly, the largest difference in total HoNOS severity ratings was observed between traumatized refugees and inpatients with anxiety disorders (ES = 1.31). Diagnostically speaking, most traumatized refugees are considered to belong to precisely this group given that they are often diagnosed with PTSD. However, the present results indicate that this may not be the most appropriate comparison. The HoNOS disability profile of the traumatized refugee outpatients is much more versatile, and the severity of disability is also much higher. This highly versatile disability profile highlights the need for a broader assessment of symptoms among traumatized refugees in general. Careful consideration needs to be taken regarding the choice of primary diagnosis and possible comorbid disorders. According to their present HoNOS profile, some of the more pertinent comorbidities in traumatized refugees may very well be cognitive problems and interpersonal problems (i.e. personality disorders), which are known to complicate treatment of PTSD [19]. Epidemiological studies have indicated that personality disorders are a frequent comorbidity in individuals with PTSD and trauma [20]. Therefore, the high level of interpersonal problems in the present group of refugees with chronic traumatization is not surprising. Finally, cognitive impairment in traumatized refugees can be related to a number of different causes, including sleep deprivation, long lasting PTSD, severe depression, dissociative disorders, mild traumatic brain injury (caused by blows to the head), and different combinations of these. These causes should ideally be carefully assessed and their impact on the ability to profit from treatment systematically evaluated.

Although there are, as yet, no appropriate diagnoses that capture the complex and chronic trauma adaptations among refugees, very broad problem profiles have been identified in the literature for individuals exposed to extreme traumatization - e.g. Disorders of Extreme Stress Not Otherwise Specified (DESNOS) [21] and Enduring Personality Change after Catastrophic Experience (F62.0) [10]. However, these problem profiles do not work well as diagnostic entities [22]. Moreover, there is no obvious treatment of choice for broad, complex trauma adaptations among refugees, and the prognosis is unknown.

### Differences in the rates of improvement on the HoNOS

While pre- to post-treatment change on the HoNOS was registered for the traumatized refugees, the rate of improvement was much smaller and more uneven compared to that of psychiatric inpatients. First, the disparities in the improvement rates between traumatized refugees and inpatients can be attributed to the acute- rather than the chronic state of the inpatients. That is, greater improvement can be expected during the stabilization phase of inpatients, who are in the acute stage of their psychiatric illness, than from the traumatized refugees, whose illness has probably reached a chronic state many years ago.

Second, firm conclusions about reasons for the low rate of improvement in traumatized refugees cannot be drawn from the current routinely collected treatment data. However, it is clear that the highly versatile disability profile of the traumatized refugees presents complex treatment challenges, which truly have to be addressed on both the biological, psychological, and social levels. One of the most important steps is probably systematic use of treatment management plans. This means actively utilizing knowledge about the level and profile of psychiatric disability in order to differentiate treatment needs, and focus on the impairments that seem the most pertinent (or in case of very severe disability, identify and strengthen resources to support change in other areas of functioning). That is, patients with problems primarily on the psychological level (i.e. psychological impairment) might best profit from psychological interventions. Those with many social and psychological problems will on the other hand probably have to resolve their social issues, before being able to profit from psychotherapeutic interventions, which primarily target individual change. Also, as a consequence of their complex bio-psycho-social status, the level of psychiatric disability in some traumatized refugees may be so high as to imply that some are not able to profit very much from outpatient treatment. In this case, referral to social psychiatric initiatives should be an option, but in reality, our experience is, that Western social psychiatric services are currently not well enough equipped to meet the needs of patients with cultural backgrounds that differ from the majority population. More focus on and systematic knowledge dissemination about the needs of traumatized refugees in Western social psychiatric services are needed. Finally, some of the worst functioning traumatized refugee patients could maybe also benefit from some psycho-pharmaceutic treatment in order to alleviate the worst symptoms of e.g. severe depression, before psychotherapeutic work can be initiated. However, it should be kept in mind that the administration of psychopharmaca to traumatized refugees is a complex process requiring appropriate specialist knowledge. Furthermore, based on the complex biopsychosocial disability profile of the traumatized refugees documented in the present study, the use of psychopharmaca is only advocated as a well-integrated supplement to the multidisciplinary treatment, not as a standalone “quick fix” solution. Finally, all these alternative treatment scenarios need to be studied within more appropriate study designs, where the benefits of the specific treatment components of a multidisciplinary treatment can be properly disentangled.

Most importantly, professionals who do not work with traumatized refugees on a daily basis should be aware that the rates of expected improvement for this psychiatric group are at present likely to be modest, even in situations where the best current treatments have been made available. Also, this group of patients has never been followed up over longer periods, and little is currently known about their prognosis.

### The practical use of the HoNOS with traumatized refugees

The use of the HoNOS as a measure of psychiatric disability among traumatized, refugee outpatients was as a whole found to be meaningful at the three CPTP departments. The instrument covers a range of problems that are typically encountered in psychiatric populations. As indicated by the present findings, traumatized, treatment-seeking refugees experience most of these problems as well.

There are a number of special concerns related to the use of the HoNOS among traumatized refugees. Firstly, due to the complexity of assessing social issues across different cultural backgrounds, the information available to clinicians at pre-treatment was found inadequate in relation to social problems (e.g. *problems with activities/daily living*, and *living conditions*). This was usually dealt with by adding questions about social problems directly to the initial assessment interview. Psychologists were also given three additional sessions in which to rate the HoNOS because the need for interpretation usually cuts the amount of information that can be acquired during a single session by one half. The use of the HoNOS among traumatized refugees would probably not have been feasible without these adjustments. Secondly, with regards to item 8 (comorbidity), it was often difficult to determine which of the patients’ comorbidities was the most central and most severe. However, this may be a general problem associated with the HoNOS, given that similar problems have been reported in relation to other populations as well [13]. The obvious contribution of the HoNOS in the context of treatment of traumatized refugees is that it provides systematic information about a range of complex biopsychosocial problems which are necessary to enable appropriate management plans and facilitate cross-disciplinary service utilization within this group. Furthermore, a practical quality of the HoNOS is also that it is an observer-rated instrument. Thus, in a refugee treatment context, it does not require translation, and can easily be employed as a routine measure. Finally, Rasch analyses of the HoNOS within the present data indicate that it is a undimensional measure of psychiatric disability with stable psychometric properties across different measurement points, and across different cultural subgroups represented in the present material (Palic, Kappel, Makranksy: Rasch validation and cross-validation of the Health of Nation Outcome Scales (HoNOS) for purposes of monitoring of traumatized refugees in Western psychiatric care, submitted).

The use of Western scales in other cultures is generally not advised unless verification of their applicability has been provided. However, most of the CPTP patients in the present study are former refugees who had been resettled in Denmark for over a decade. Thus, although the traumatized refugees may not be well integrated into the Danish way of life, the same societal responsibilities and expectations as all other citizens and psychiatric patients are placed upon them. In this case, it is necessary to evaluate the psychiatric disability (including social function) of traumatized refugee patients in relation to the role expectations of the society in which they live. Failure to do so raises the risk of traumatized former refugees “falling through” the cracks of the treatment- and social systems, and not getting the necessary help and support to maintain a worthy existence.

### Limitations

As already mentioned, the possible bias associated with the present HoNOS ratings due to the inpatient vs. outpatient setting, cannot be evaluated in the present study. Also, inter-rater agreement on the HoNOS was not assessed at CPTP. All this contributes to some uncertainty regarding the HoNOS ratings among the traumatized refugees. On the other hand, the present comparison across treatment settings is justified by previous research. A direct comparison cannot be made between the levels of comorbidity in the traumatized refugees and psychiatric inpatient groups, because the first were rated on comorbidity and the latter on anxiety. However, the levels of comorbidity/anxiety can be understood as reflecting the most pertinent comorbidity problems associated with each group. The present disability levels were recorded at only one hospital and at three departments of a specialized refugee clinic. However, given that the hospital and the refugee clinic are part of the Danish national mental health system, to which everyone in general has equal access, the selection bias associated with the presented disability levels is likely to be small.

## Conclusions

The present results indicate that systematic assessment of bio-psycho-social outcomes in traumatized treatment seeking refugees in Western psychiatric care is very important. High levels of complex psychiatric comorbidity and social impairment registered on the HoNOS indicate that appropriate treatment and management plans can be difficult to devise without systematic evaluation of the combined load of bio-psycho-social problems in this group. Access to appropriate services across the psychiatric, social, and health care systems can also be hampered due to the undescribed nature of the complex treatment needs of traumatized refugees. In our experience, the HoNOS lends itself well to adapted use with traumatized refugees in outpatient treatment. Future studies should further qualify the validity of the HoNOS in the traumatized refugees by exploring whether patients’ status on the HoNOS is predictive of long-term adjustment and differential outcomes.

### Ethical standards

The article analyzes data collected routinely for each patient as a part of the everyday practice and treatment. Data was collected with the approval of Aarhus University Hospital and Psychiatric Center North Zealand. Data are kept in the accordance with Danish law about personal data protection and the study was reported to the Danish Data Protection Agency. According to the Danish National Committee on Health Research Ethics, no further ethics approvals are required in Denmark when biomedical material is not included in the study. Also, in such instances informed consent from the participants is not requested.
